# MicroRNA-disease association prediction by matrix tri-factorization

**DOI:** 10.1186/s12864-020-07006-x

**Published:** 2020-11-18

**Authors:** Huiran Li, Yin Guo, Menglan Cai, Limin Li

**Affiliations:** School of Mathematics and Statistics, Xi’an Jiaotong University, Xianning West 28, Xi’an, China

**Keywords:** micoRNA-disease association prediction, Matrix tri-factorization

## Abstract

**Background:**

Biological evidence has shown that microRNAs(miRNAs) are greatly implicated in various biological progresses involved in human diseases. The identification of miRNA-disease associations(MDAs) is beneficial to disease diagnosis as well as treatment. Due to the high costs of biological experiments, it attracts more and more attention to predict MDAs by computational approaches.

**Results:**

In this work, we propose a novel model MTFMDA for miRNA-disease association prediction by matrix tri-factorization, based on the known miRNA-disease associations, two types of miRNA similarities, and two types of disease similarities. The main idea of MTFMDA is to factorize the miRNA-disease association matrix to three matrices, a feature matrix for miRNAs, a feature matrix for diseases, and a low-rank relationship matrix. Our model incorporates the Laplacian regularizers which force the feature matrices to preserve the similarities of miRNAs or diseases. A novel algorithm is proposed to solve the optimization problem.

**Conclusions:**

We evaluate our model by 5-fold cross validation by using known MDAs from HMDD V2.0 and show that our model could obtain the significantly highest AUCs among all the state-of-art methods. We further validate our method by applying it on colon and breast neoplasms in two different types of experiment settings. The new identified associated miRNAs for the two diseases could be verified by two other databases including dbDEMC and HMDD V3.0, which further shows the power of our proposed method.

## Background

MicroRNAs(miRNAs), a class of small, endogenous, non-coding RNAs including approximately 22 nucleotides, could regulate post-transcription of gene expression and RNA silencing by binding specific target messenger RNAs through base-pairing interactions [1, 2]. Since the first miRNA named lin-4 was found twenty years ago by Victor Ambros [3], with the development of technology, an increasing number of studies found that miRNAs play important roles in various stages of biological processes [3], such as cell development [4], proliferation [5] and viral infection [6]. Meanwhile, biological experiments indicate that miRNAs are involved in close relationships with the emergence and development processes of various human diseases [7]. For example, the study in [8] showed that a chromosomal translocation at 12q5 could influence the expression of let-7 and finally could cause the repress of the oncogene High Mobility Group A2(Hmga2). Another example is that mir-7 could influence epidermal growth factor receptor (EGFR) expression and protein kinase B activity in head and neck cancer(HNC) [9]. Furthermore, the work in [10] showed that mir-15a is a potential marker to differentiate between benign and malignant renal tumors in biopsy and urine samples. It is very important to identify miRNA-disease associations (MDAs) for the research on disease mechanism and discovering disease biomarkers. Due to the high costs of the current biological technologies, computational methods are useful by prioritizing candidate miRNAs for specific diseases.

The main information used for predicting MDAs mainly includes miRNA similarities, disease similarities and the known MDAs. Generally, miRNA similarities could be computed by using functional or sequence information of miRNAs, disease similarities could be obtained by using the phenotype terms, and the known MDAs could be obtained from databases such as HMDD [11]. The main challenge in MDA prediction is how to optimally utilize these information and predict MDAs with a high accuracy. Based on these information, many computational models are proposed to predict new MDAs.

The existing methods follow two lines. The first line is to determine the link probabilistically by using random walk. For example, RWRMDA [12] adopts random walk on the miRNA functional similarity network. It first gives each miRNA an initial probability in the miRNA functional similarity network(MFSN), and then use a random walk algorithm until the probability get stable. However, this method cannot predict new disease without any known related miRNAs. Thus Shi et al. [13] use the random walk algorithm on miRNA target and disease genes at the same time to map the protein-protein interaction (PPI) network, and then they construct a bipartite miRNA-disease network by using *p*-values in the PPI network and identify co-regulated modules by hierarchical clustering analysis. Later Xuan et al. [14] develop the MIDP method by using the prior information of nodes. They first divide the diseases related to the miRNAs into labeled nodes and unlabeled nodes, and establish the transition matrices for the two categories of nodes. Then by using the random walk algorithm on the two weighted transition matrices, the final miRNA ranking could be obtained. Liu et al. [15] proposed a random walk method to predict the associations by combining the multiple data sources.

The second line is to formulate the problem as machine learning problems such as classification, matrix completion. For classification formulation, examples include the RLSMDA [16] and the MTDN [17] methods. RLSMDA [16] develops Regularized Least Squares algorithm by training two classifiers from the miRNA space and the disease space. However, how to choose the parameter of RLSMDA and how to combine the classifiers need to be studied furthermore. Xu et al. [17] introduce the MTDN approach based on miRNA target-dysregulated network to prioritize novel disease miRNAs. The method first constructs the network by combining computational target prediction with miRNA and mRNA expression profiles in tumor and non-tumor tissues, and then applies a support vector machine classifier to distinguish positive miRNA-disease associations from negative ones by extracting the feature of network topologic information. However, it is hard to obtain the negative miRNA-disease associations. Another option using machine learning is matrix completion such as MCLPMDA [18], IMCMDA [19], CMFMDA [20] and PMAMCA [21]. MCLPMDA [18] constructs new miRNA and disease similarity matrices by matrix completion algorithm firstly, and then uses label propagation algorithm to predict miRNAs. Chen et al. [19] propose a method named IMCMDA based on nonnegative matrix factorization, whose main idea is to complete the missing miRNA-disease association based on the known associations and miRNA and disease similarity. CMFMDA [20] and PMAMCA [21] both factorize the association matrix into two matrices which representing the features for miRNAs and diseases, respectively.

In this study, we propose a novel computational method MTFMDA to predict new MDAs by matrix tri-factorization, to follow the idea of matrix completion. The main idea of MTFMDA is that we factorize the complete MDA matrix to three matrices, a feature matrix *P* for miRNAs, a feature matrix *Q* for diseases, and a low-rank matrix *D* representing relationships between miRNA features and disease features. Laplacian regularizers are used for the feature matrices *P* and *Q* by using two types of miRNA similarities, and two types of disease similarities, respectively. Optimal matrices *P*,*D* and *Q* are learnt by using the known MDAs and the Laplacian regularizers, and then the MDA matrix is completed by *PDQ*^*T*^ and thus new MDAs can be identified.

The contributions in this work are listed as follows:

- We propose a new MDA prediction model by matrix tri-factorization model, which combines the two types of miRNA similarities, two types of disease similarities, and the known miRNA-disease associations, and predict new MDAs by completing the MDA matrix. We develop an algorithm for solving the optimization problem.
- We evaluate our MTFMDA model by 5-fold cross-validation and obtain higher accuracies than other state-of-art methods.
- We apply our method on two diseases to identify related miRNAs, and our prediction results could be supported by other databases. This further validates the effectiveness of our model MTFMDA.

## Materials and methods

### Datasets

#### Human miRNA-disease associations

We collect the known human miRNA-disease associations from HMDD V2.0 database (June, 2014) [11], and obtain 3693 associations among 368 miRNAs and 383 diseases.

#### MiRNA functional similarity and sequence similarity

The functional similarities among miRNAs can be calculated by the method proposed in [22], and we download the similarity data from http://www.cuilab.cn. Since miRNA’s function is closely relevant to the miRNA sequence [23], we also obtain the miRNA sequence similarity from http://www.mirbase.org/ftp.shtml. The integrated similarities among miRNAs are defined as the average of the functional similarity and the sequence similarity, and the integrated similarity matrix for miRNAs is denoted as *S*_*m*_.

#### Two disease semantic similarities

To calculate disease semantic similarities, Wang [22] and Xuan [24] propose two methods based on the Medical Subject Headings (MeSH) descriptors which could be downloaded from the National Library of Medicine (http://www.nlm.nih.gov/).

Wang’s method [22] first calculates the semantic value and contribution value of a disease, and then uses these two values to compute the semantic similarity between two diseases. Unlike Wang’s method, Xuan et al. [25] improves the calculation method of semantic value. It also uses semantic value and contribution value to calculate the semantic similarity. We use the integrated similarity in our work by averaging the two types of semantic similarities, and denote the integrated similarity matrix for diseases as *S*_*d*_.

### Our proposed method via matrix tri-factorization

#### Problem statement and notations

We are now given the integrated similarity matrix $\phantom {\dot {i}\!}S_{m}\in R^{n_{m}\times n_{m}}$ among *n*_*m*_ miRNAs $\phantom {\dot {i}\!}\left \{m_{1},\cdots,m_{n_{m}}\right \}$, and the integrated similarities $\phantom {\dot {i}\!}S_{d}\in R^{n_{d}\times n_{d}}$ among *n*_*d*_ diseases $\left \{d_{1},\cdots,d_{n_{d}}\right \}$. We are also given the miRNA-disease association (MDA) indicator matrix $\phantom {\dot {i}\!}A\in R^{n_{m}\times n_{d}}$ defined as follows

$$\begin{aligned} A(i,j)= \left\{\begin{array}{ll} 1,& {i}-\text{th miRNA } m_{i} \text{ is associated with } {j}-\text{th disease } d_{j}, \\ 0,& \text{association between } {i}-\text{th miRNA } m_{i} \text{ and } {j}-\text{th disease } d_{j} \text{ is unknown.} \end{array} \right. \end{aligned} $$

We denote *Ω*={(*i*,*j*)|*A*_*ij*_=1} to be the indices for the miRNA-disease pairs which are known to be associated, and *Ω*^*c*^={(*i*,*j*)|*A*_*ij*_=0} to be all the pairs whose associations are unknown. For any matrix *M*, we denote $\mathcal {R}_{\Omega }(M)$ by only keeping its *Ω* part and forcing its *Ω*^*c*^ part to be zeros, that is,

$$\mathcal{R}_{\Omega}(M)_{{ij}}= \left\{\begin{array}{lc} M_{{ij}},~~~if (i,j)\in\Omega\\ 0,~~~~~~if (i,j)\in\Omega^{c}. \end{array}\right. $$

Our aim in this work is to complete the *Ω*^*c*^ part in matrix *A*, and recover the complete matrix $\tilde {A}$.

#### MTFMDA model

**Fig. 1:**
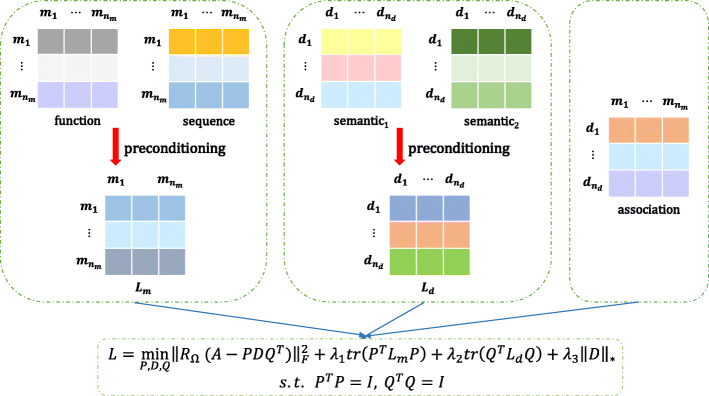
Flowchart of MTFMDA model to infer the potential miRNA-disease associations

We propose our MTFMDA method by considering the following three aspects. First, the unknown complete miRNA-disease association (MDA) matrix $\tilde {A}$ can be factorized into three matrices, a feature matrix for miRNAs $\phantom {\dot {i}\!}P\in R^{n_{m}\times r_{m}}$, a feature matrix for diseases $\phantom {\dot {i}\!}Q\in R^{n_{d}\times r_{d}}$, and the feature relationship matrix $\phantom {\dot {i}\!}D\in R^{r_{m}\times r_{d}}$. The factorization $\tilde {A} = PDQ^{T}$ implies that the column vectors in $\tilde {A}$ lie in the subspace spanned by the column vectors in *P*, and the row vectors in $\tilde {A}$ lie in the subspace spanned by the column vectors in *Q*. *D* is generally required to be low rank, and *P* and *Q* are orthonormal matrices satisfying *P*^*T*^*P*=*I* and *Q*^*T*^*Q*=*I*. Second, the complete $\tilde {A}$ should recover the known associations between miRNAs and diseases, i.e, the *Ω* part of the difference matrix $\left (A-\tilde {A}\right)$ should be zero or as small as possible. Third, the feature vectors in *P* and *Q* should preserve the similarity information hidden in the *S*_*m*_ and *S*_*d*_, respectively, and thus two Laplacian regularizers should be used for preserving the geometric structure. By considering the above three aspects, we propose the following MTFMDA model

1 $$ \begin{aligned} &\underset{P,D,Q}{\min} \left\|\mathcal{R}_{\Omega}\left(A-P D Q^{T}\right)\right\|^{2}_{F} +\lambda_{1} tr\left(P^{T} L_{m} P\right) +\lambda_{2} tr\left(Q^{T} L_{d} Q\right) +\lambda_{3}\|D\|_{*}\\ &s.t.\quad P^{T} P=I, Q^{T} Q=I, \end{aligned}  $$

where *λ*_1_,*λ*_2_ and *λ*_3_ are the regularization parameters to control the trade-offs. The first term is to recover the known MDAs in *A*. In the second term, *L*_*m*_=*D*_*m*_−*S*_*m*_ is the Laplacian matrix for the miRNAs, where *D*_*m*_ is a diagonal matrix with the *i*-th diagonal element being the sum of *i*-th row in *S*_*m*_. In the third term, *L*_*d*_ is the Laplacian matrix for diseases, defined in the same way as *L*_*m*_. Once the optimal *P*,*D* and *Q* are solved in the optimization problem, the completed MDA matrix $\tilde {A}$ can be obtained by $\tilde {A}=PDQ^{T}$. The flowchart of our method is shown in Fig.1.

#### Optimization algorithm

In order to solve optimization problem above, we develop an alternate iteration algorithm to update *P*, *D* and *Q* alternately.

**Step 1**: Fix *P* and *Q*, solve *D*By fixing *P* and *Q* in the optimization problem (1), the sub-problem to solve *D* can be obtained as follows:

2 $$ \begin{aligned} \underset{D}{\min} \left\|\mathcal{R}_{\Omega}\left(A-P D Q^{T}\right)\right\|^{2}_{F}+\lambda_{3}\|D\|_{*}.\\ \end{aligned}  $$

The sub-problem can be solved by an accelerated gradient descent algorithm [26] with the following iterations,

3 $$ \begin{aligned} &Y_{k}=D_{k}+\gamma_{k}\left(\gamma_{k-1}^{-1}-1\right)\left(D_{k}-D_{k-1}\right),\\ &D_{k+1}=\arg\min\nolimits_{D} \lambda_{3}\left|\left|D\left|\left|{~}_{*}+\frac{s}{2}\right|\right|D-\left(Y_{k}-\frac{1}{s}\bigtriangledown f\left(Y_{k}\right)\right)\right|\right|^{2}_{F},\\ &\gamma_{k+1}=\left(\sqrt{\gamma^{4}_{k}+4\gamma^{2}_{k}}-\gamma^{2}_{k}\right)/2, \end{aligned}   $$

where *s* is a proximal parameter for estimating the second-order gradient of *f*(*Y*). The second equation in (3) can be solved by using the linearized Bregman iteration as a special form of Uzawa’s algorithm proposed in Cai et al. [27].

**Step 2**: Fix *D* and *Q*, solve *P*.

By fixing *D* and *Q* in optimization problem (1), we obtain the sub-problem of *P* as follows:

4 $$ \begin{aligned} \underset{P}{\min} \left\|\mathcal{R}_{\Omega}\left(A-P D Q^{T}\right)\right\|^{2}_{F}+\lambda_{1} tr\left(P^{T} L_{m} P\right).\\ \end{aligned}  $$

Similarly to solving *D* in step 1, we could also use the accelerated gradient descent (APG) model to update *P* as follows:

5 $$ \begin{aligned} &\hat{Y}_{k}=P_{k}+\gamma_{k}\left(\gamma_{k-1}^{-1}-1\right)\left(P_{k}-P_{k-1}\right),\\ &P_{k+1}=\arg\min\nolimits_{P} \lambda_{1} tr\left(P^{T} L_{m} P\right)+\frac{s}{2}\left|\left|P-\left(\hat{Y}_{k}-\frac{1}{s}\bigtriangledown f\left(\hat{Y}_{k}\right)\right)\right|\right|^{2}_{F},\\ &\gamma_{k+1}=\left(\sqrt{\gamma^{4}_{k}+4\gamma^{2}_{k}}-\gamma^{2}_{k}\right)/2. \end{aligned}   $$

Wen’s algorithm proposed in [28] is used to solve the second Eq. in (5).

**Step 3**: Fix *D* and *P*, solve *Q*.

By fixing *D* and *P* in optimization problem (1), we obtain the sub-problem of *Q* as follows:

6 $$ \begin{aligned} \underset{Q}{\min} \left\|\mathcal{R}_{\Omega}\left(A-P D Q^{T}\right)\right\|^{2}_{F}+\lambda_{2} tr\left(Q^{T} L_{d} Q\right).\\ \end{aligned}  $$

It can be seen that the sub-problem (6) to solve for *Q* is the same with the sub-problem (4) to solve for *P*. Thus we skip the details.

Overall, the framework of our algorithm is shown as follows:


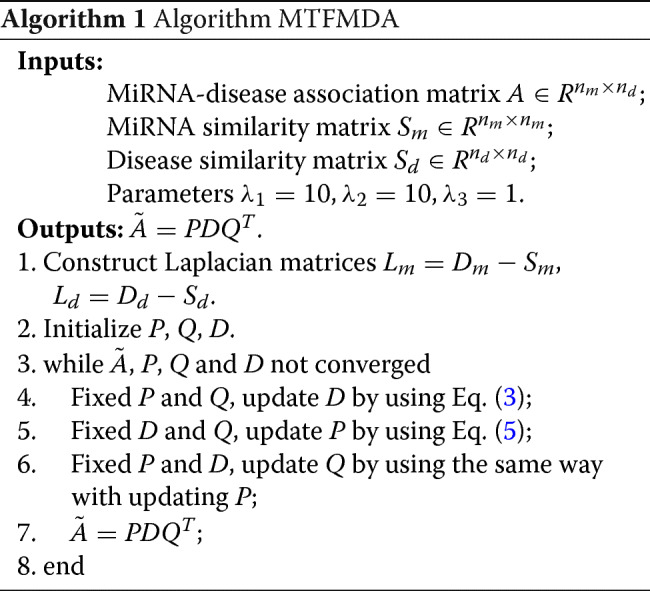


## Results

In this section, we will first evaluate our method on the known associations collected from HMDD V2.0 database by 5-fold cross validation. Then we further evaluate our method by using the probability of recovering a true association in the top-*t* predictions for new diseases. We also estimate the contribution of the performance for the known association matrix, integrated miRNA and disease similarity.

### Comparing methods

We compare our MTFMDA method with the following seven methods.

- **RLSMDA** [16].The model is a semi-supervised learning method, and it develops a Regularized Least Squares algorithm by training two classifiers from the miRNA space and the disease space (we use parameter *ω*=0.9).
- **RWRMDA** [12]. The model is a random walk method which infers potential miRNA-disease interactions by implementing random walk on the miRNA-miRNA functional similarity network (we use parameters *r*=0.2, threshold = 10^−6^).
- **IMCMDA** [19]. The method is a matrix completion model by nonnegative matrix factorization using the same datasets with our method (we use parameter *r*=100).
- **NCPMDA** [29]. The method is a non-parametric universal network-based method that combines miRNA space and disease space.
- **KBMFMDA** [30]. The model combines kernel-based nonlinear dimensionality reduction, matrix factorization and binary classification. The main idea of the method is to project miRNAs and diseases into a subspace and estimate the association network in the subspace.
- **CMFMDA** [20]. The model factorizes the association matrix into two parts which represent miRNA and disease information, respectively. SVD factorization is used to initialize the two parts.
- **PMAMCA** [21]. The method divides the association matrix into two latent matrices, and solve the matrix factorization by using the recommend system. Note that this method doesn’t use miRNA and disease similarity matrices.

### Evaluating our method by cross-validation

We first evaluate the performance of our MTFMDA method by the 5-fold cross validation framework. We use the data with 3693 associations between 368 miRNAs and 383 diseases collected from the HMDD V2.0 database. For the 5-fold cross validation, we divide 368 miRNAs into five folds. We take one fold as the test set, and take the rest as the training set. Each fold is taken as the test set once in turn. After obtaining the complete MDA matrix $\tilde {A}$, we rank the scores for all the test pairs of miRNA-diseases. If the rank of an miRNA-disease pair exceeds a given threshold, then the pair is considered to have an association. In our method, we set the parameters as *λ*_1_=*λ*_2_=10 and *λ*_3_=1. The dimension parameters *r*_*m*_ and *r*_*d*_ are set as the one sixth of *n*_*m*_ and *n*_*d*_, respectively.

**Fig. 2:**
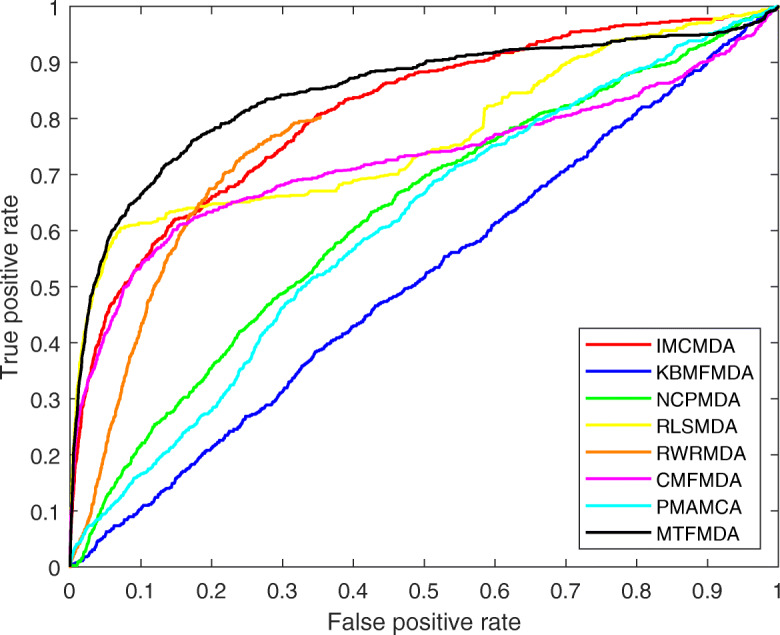
Performance comparisons between our method MTFMDA and baseline methods(IMCMDA, KBMFMDA, NCPMDA, RLSMDA, RWRMDA, CMFMDA, PMAMCA) in terms of AUC based on 5-fold cross validation

**Fig. 3:**
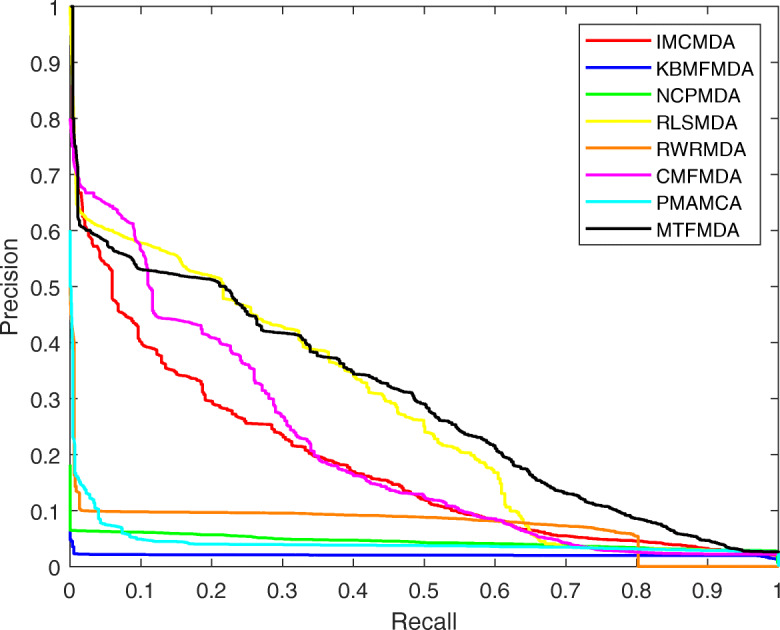
Performance comparisons between MTFMDA and baseline methods(IMCMDA, KBMFMDA, NCPMDA, RLSMDA,RWRMDA, CMFMDA, PMAMCA) in terms of PR curve

We first plot Receiver Operation Characteristics (ROC) curves for all the methods to check the true positive rates and false positive rates. In the ROC curve, the x-axis is the true positive rate (TPR) and the y-axis is the false positive rate (FPR). The ROC curves for the all the methods are plotted in Fig. 2. We can see that our MTFMDA could obtain the best ROC curve. We then perform 50 runs of 5-fold cross validation, and calculate the AUC (area under curve) values. The average AUC values of MTFMDA, RLSMDA, RWRMDA, IMCMDA, NCPMDA, KBMFMDA, CMFMDA and PMAMCA are reported in Table 1. The results show that our method achieves the highest AUC value and performs better than other methods. We further analyze the differences of inference capability between our method and others. Note that for each method we obtain 50 AUC values for the 50 runs of 5-fold cross validation. Thus, the paired t-test can be used to check whether our method is significantly better than other methods. The *p*-values between our method and other five methods are reported in Table 1. The results show that our method MTFMDA performs significantly better than other methods. We further plot the Precision-Recall curves in Fig. 3 for all the methods, and we can also see that our method performs better than all the other comparing methods.

**Table 1:** Performance comparisons between our method MTFMDA and baseline methods in terms of AUCs based on 5-fold cross validation and paired t-test *p*-values which compare our method with other methods

	IMCMDA	KBMFMDA	NCPMDA	RLSMDA	RWRMDA	CMFMDA	PMAMCA	MTFMDA
AUC	0.8114	0.5110	0.6263	0.7657	0.7778	0.7502	0.6061	0.8484
*p*-value	8.43e-94	7.87e-196	1.36e-167	7.76e-125	1.88e-119	8.11e-046	2.99e-57	

### Probability to recover true associated miRNAs for new diseases

**Fig. 4:**
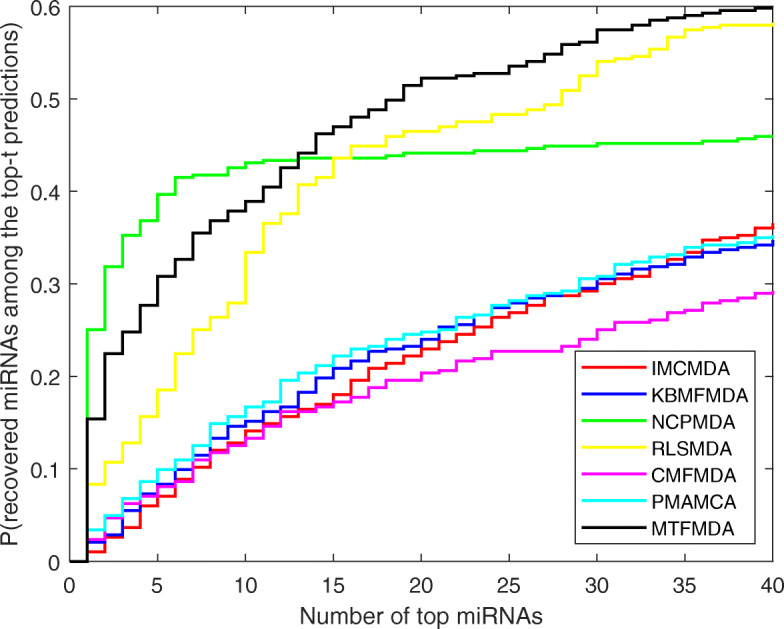
Performance comparisons between MTFMDA and baseline methods(IMCMDA, KBMFMDA, NCPMDA, RLSMDA, CMFMDA, PMAMCA) in predicting potential miRNAs for new diseases

We further evaluate our MTFMDA method by the probability of recovering a true association in the top-*t* predictions for a new disease. The probability can measure whether the method can predict potential related miRNA for a new disease. The measurement has been used in many other publications such as [19, 31, 32]. In detail, for each test disease, we first mask its known associated miRNAs as zero in matrix *A*, and then apply our model to obtain the ranks of the masked true associated miRNAs. Thus for all the 383 diseases, we could obtain the ranks of the true associated miRNAs among all the miRNAs. We could then plot the cumulative distribution function (CDF), where x-axis represents the top-*t* predicted miRNAs, and y-axis represents the probability of recovering a true association in the top *t* predictions. Other methods could also plot the curves, except the RWRMDA, which cannot predict the new diseases. The CDFs for the five methods are shown in Fig. 4. From the figure, we can see that though NCPMDA performs better than ours for the top 13 miRNAs, the probability of recovering a true association in the top *t* predictions does not change much when *t* from 13 to 40. When *t* is from 13 to 40, our method could recover true associated miRNAs with highest probabilities.

### Contributions of different data sources

We use three data sets in our model, miRNA similarity *S*_*m*_, disease similarity *S*_*d*_ and known miRNA-disease association matrix *A*. To examine their contributions to the performance of our model, we first change each of the three matrices to a random matrix, and then apply our MTFMDA model to check the AUC values based on 5-fold cross validation. If one data type contributes the most, then the corresponding AUC should decrease a lot when changing to the data to be random. We change *S*_*m*_,*S*_*d*_ and *A* to be random in turn, and the resulting AUCs are reported in Table 2. As shown in Table 2, the average AUC value based on the random miRNA similarity matrix is much lower than the other two types in our model, and thus the miRNA similarity contributes the most to the performance of our model. We also see that the disease similarity contributes the least in our MTFMDA model.

**Table 2:** AUCs of our model by changing one of the three data sources to be random in turn

Randomized Data sources	Average value of AUCs
Known miRNA-disease associations	0.5025
MiRNA similarities	0.1951
Disease similarities	0.8360

## Discussion

We apply two diseases including colon and breast neoplasms to verify the effectiveness of the MTFMDA for miRNA-disease association prediction. For each disease, we apply our MTFMDA model to predict the top-*t* associated miRNAs, and then examine these predictions by two datasets: dbDEMC [33] and HMDD V3.0 [34].

Table 3 shows the predicted top 50 miRNAs for the colon neoplasms using our MTFMDA model. Colon neoplasms is an out-of-control cell growth, and it is the second leading cause of death in cancer and the most common tumor in the gastrointestinal tract [35, 36]. Many factors will cause the neoplasms, such as old age, unhealthy lifestyle and heredity [37]. Recently, more and more evidence proved that some miRNAs are related to the colon neoplasms. For example, Zhang et al. [38] showed that mir-21, mir-17 and mir-19a promote the metastasis and spread of colon neoplasms. Coincidentally, the expression levels of miR-106a of normal human are higher than colon cancer patients [39]. Shi et al. [40] found that mir-145 could down-regulate the IRS-1 protein in the colon neoplasms cells and inhibit cells growth through targeting the IRS-1 3’-untranslated region. From our prediction results shown in Table 3, we can see that all the predicted top 10 miRNAs by our method can be confirmed by both the dbDEMC and HMDD V3.0 databases, and 46 among the top 50 miRNAs can be confirmed by dbDEMC and HMDD V3.0 databases. This validates the effectiveness of our MTFMDA method.

**Table 3:** The predicted top 50 miRNAs associated with colon neoplasms and the evidence from databases HMDD V3.0 and dbDEMC

miRNA	Evidence	miRNA	Evidence
hsa-mir-155	dbDEMC;HMDD	hsa-let-7b	dbDEMC;HMDD
hsa-mir-146a	dbDEMC;HMDD	hsa-mir-143	dbDEMC;HMDD
hsa-mir-328	dbDEMC;HMDD	hsa-mir-200a	HMDD
hsa-mir-29a	dbDEMC;HMDD	hsa-mir-195	dbDEMC;HMDD
hsa-mir-17	dbDEMC;HMDD	hsa-let-7c	dbDEMC;HMDD
hsa-mir-34a	dbDEMC;HMDD	hsa-mir-326	dbDEMC;HMDD
hsa-mir-20a	dbDEMC;HMDD	hsa-mir-23a	dbDEMC;HMDD
hsa-mir-15a	dbDEMC;HMDD	hsa-mir-210	dbDEMC;HMDD
hsa-mir-221	dbDEMC;HMDD	hsa-mir-141	dbDEMC;HMDD
hsa-mir-145	dbDEMC;HMDD	hsa-mir-320a	dbDEMC
hsa-mir-19a	dbDEMC;HMDD	hsa-let-7i	dbDEMC;HMDD
hsa-mir-206	dbDEMC	hsa-mir-214	dbDEMC
hsa-mir-29c	dbDEMC	hsa-let-7d	dbDEMC;HMDD
hsa-mir-593	unconfirmed	hsa-mir-34b	unconfirmed
hsa-mir-150	dbDEMC;HMDD	hsa-mir-133b	dbDEMC;HMDD
hsa-mir-18a	dbDEMC;HMDD	hsa-mir-146b	dbDEMC
hsa-mir-222	dbDEMC;HMDD	hsa-let-7e	dbDEMC;HMDD
hsa-mir-15b	dbDEMC;HMDD	hsa-mir-663a	dbDEMC
hsa-mir-142	HMDD	hsa-mir-200c	HMDD
hsa-mir-223	dbDEMC;HMDD	hsa-mir-148a	dbDEMC;HMDD
hsa-mir-200b	dbDEMC;HMDD	hsa-mir-193a	unconfirmed
hsa-mir-483	HMDD	hsa-mir-574	dbDEMC
hsa-mir-30b	dbDEMC;HMDD	hsa-mir-106a	dbDEMC;HMDD
hsa-mir-34c	unconfirmed	hsa-let-7g	dbDEMC;HMDD
hsa-mir-106b	dbDEMC;HMDD	hsa-mir-335	dbDEMC;HMDD

For the breast neoplasms, we evaluate our method in another way. We mask all the known associated miRNAs with breast neoplasms and apply our MTFMDA method to obtain the predicted top 50 associated miRNAs for the breast neoplasms, shown in Table 4. From this table, we can see that, all the top 10 miRNAs are confirmed by the two databases, and 49 of the top 50 miRNAs can be confirmed by the two databases. Through the Table 4, we found that hsa-mir-155 ranks the first, and it has been found that this miRNA could affect many cancers in recent studies, such as breast neoplasms, colon neoplasms and esophageal neoplasms [41–43].

**Table 4:** The predicted top 50 miRNAs associated with breast neoplasms and the evidence from databases HMDD V3.0 and dbDEMC

miRNA	Evidence	miRNA	Evidence
hsa-mir-155	dbDEMC;HMDD	hsa-mir-106b	dbDEMC;HMDD
hsa-mir-146a	dbDEMC;HMDD	hsa-mir-143	dbDEMC;HMDD
hsa-mir-328	dbDEMC;HMDD	hsa-let-7c	dbDEMC;HMDD
hsa-mir-29a	dbDEMC;HMDD	hsa-mir-195	dbDEMC;HMDD
hsa-mir-15a	dbDEMC;HMDD	hsa-mir-326	dbDEMC;HMDD
hsa-mir-17	dbDEMC;HMDD	hsa-mir-141	dbDEMC;HMDD
hsa-mir-20a	dbDEMC;HMDD	hsa-let-7i	dbDEMC;HMDD
hsa-mir-145	dbDEMC;HMDD	hsa-let-7d	dbDEMC;HMDD
hsa-mir-34a	dbDEMC;HMDD	hsa-mir-23a	dbDEMC;HMDD
hsa-mir-221	dbDEMC;HMDD	hsa-mir-210	dbDEMC;HMDD
hsa-mir-19a	dbDEMC;HMDD	hsa-mir-34b	dbDEMC;HMDD
hsa-mir-206	dbDEMC;HMDD	hsa-let-7e	dbDEMC;HMDD
hsa-mir-29c	dbDEMC;HMDD	hsa-mir-335	dbDEMC;HMDD
hsa-mir-150	dbDEMC;HMDD	hsa-mir-146b	HMDD
hsa-mir-18a	dbDEMC;HMDD	hsa-mir-214	dbDEMC;HMDD
hsa-mir-142	HMDD	hsa-mir-200c	dbDEMC;HMDD
hsa-mir-15b	dbDEMC;HMDD	hsa-mir-320a	dbDEMC;HMDD
hsa-mir-200b	dbDEMC;HMDD	hsa-let-7g	dbDEMC;HMDD
hsa-mir-222	dbDEMC;HMDD	hsa-mir-133b	dbDEMC;HMDD
hsa-mir-223	dbDEMC;HMDD	hsa-mir-106a	dbDEMC;HMDD
hsa-let-7b	dbDEMC;HMDD	hsa-mir-451a	dbDEMC;HMDD
hsa-mir-34c	dbDEMC;HMDD	hsa-mir-193a	dbDEMC;HMDD
hsa-mir-30b	dbDEMC;HMDD	hsa-mir-663a	HMDD
hsa-mir-200a	dbDEMC;HMDD	hsa-mir-152	dbDEMC;HMDD
hsa-mir-483	unconfirmed	hsa-mir-92b	dbDEMC;HMDD

Overall, the case studies on colon and breast neoplasms further validate the effectiveness of our MTFMDA method for predicting miRNA-disease associations.

## Conclusion

Identifying potential miRNA-disease associations could help understand the pathogenesis of the disease from a genetic perspective. In this work, we propose a computational method MTFMDA to predict new MDAs by using an idea of matrix tri-factorization. Different from other matrix completion methods, we factorize the complete MDA matrix to three matrices including a feature matrix for miRNAs, a feature matrix for diseases and a low-rank matrix representing the relationships between miRNA features and disease features. Experiments show that our method performs better for predicting miRNAs associated with new diseases. As we have shown, based on the 5-fold cross validation, the comparisons on the ROC curves, AUCs and Precision-Recall curves show that our MTFMDA performs better than the other methods. Furthermore, the experiments to predict associated miRNAs for colon and breast neoplasms also demonstrate the effectiveness of our method. However, this research only takes the average of two types of similarities of miRNAs and diseases, but not consider how to combine the two similarities optimally. This could be our future topic to work on.

## Data Availability

Human miRNA-disease associations were downloaded from HMDD V2.0 database [11]. MiRNA functional similarity was downloaded from http://www.cuilab.cn. MiRNA sequence data was downloaded from http://www.mirbase.org/ftp.shtml. Disease semantic similarity were downloaded from National Library of Medicine http://www.nlm.nih.gov.

## Abbreviations

miRNA: MicroRNA
MDA: MicroRNA disease association
MTFMDA: MicroRNA disease association prediction by matrix tri-factorization
HNC: Head and neck cancer
PPI: Protein-protein interaction network
HMDD V2.0: Human microRNA disease database version 2.0
MeSH: Medical subject headings
CDF: Cumulative distribution function
IRS: Insulin receptor substrate
dbDEMC: Database of differentially expressed miRNAs in human cancers
